# Novel Insights into the Role of Caveolin-2 in Cell- and Tissue-Specific Signaling and Function

**DOI:** 10.1155/2011/809259

**Published:** 2011-12-20

**Authors:** Grzegorz Sowa

**Affiliations:** Department of Medical Pharmacology and Physiology, University of Missouri, Columbia, MO 65212, USA

## Abstract

Caveolin-2 is one of the major protein components of cholesterol- and glycosphingolipid-rich flask-shaped invaginations of plasma membrane caveolae. A new body of evidence suggests that caveolin-2 plays an important, and often more direct, role than caveolin-1 in regulating signaling and function in a cell- and tissue type-specific manner. The purpose of this paper is to primarily focus on discussing how these recent discoveries may help better understand the specific contribution of caveolin-2 to lipid raft- and caveolae-regulated cell/tissue-specific signaling and functions.

## 1. Introduction

Caveolins are critical membrane proteins of cholesterol lipid-rich lipid rafts and caveolae. Three members are known within the caveolin protein family: caveolin-1 (Cav-1), Cav-2, and Cav-3 [1]. Cav-1 and -2 are ubiquitously coexpressed, while Cav-3 is muscle-specific [1]. Cav-2 tightly interacts with Cav-1 and forms hetero-oligomeric complexes within caveolae [2, 3]. The interaction with Cav-1 is necessary for transport of Cav-2 to the cell surface [4, 5]. In the absence of Cav-1, Cav-2 is degraded, and its expression is strikingly reduced [6, 7]. Caveolins play numerous important functions. In addition to being key structural proteins that organize caveolar structures, caveolin proteins are important in regulating various aspects of cell signaling and function [1, 8–11]. Although relative to Cav-1, the functional role of Cav-2 is poorly defined, and recent studies have started to reveal a growing body of evidence suggesting tissue/cell-specific role for Cav-2. This paper discusses the involvement of Cav-2 in signaling and function, with a particular emphasis of how Cav-2 may contribute to tissue- and cell-specific function of lipid raft and caveolar microdomains.

## 2. Cell Type-Specific Role for Cav-2 in Caveolae Formation and Turn Over

Unlike the indispensable role for Cav-1 in caveolae assembly in nonmuscle cells and the respective role of Cav-3 in muscle cells, the role of Cav-2 is less clear. Caveolae have been identified in ultrathin sections from lung capillary endothelium and in perigonadal adipose tissue of Cav-2 knockout (KO), suggesting that Cav-2 is not necessary for caveolae formation, at least, in the aforementioned tissues [12]. However, a more thorough examination of other organs and tissues may be required to determine the overall distribution of caveolae in Cav-2 KO mice. Most importantly, it remains to be established if and how the function of Cav-2-devoid caveolae may be altered relative to WT. In contrast to the observations made in Cav-2 KO mice, there are several studies independently indicating supportive or essential role of Cav-2 in the caveolae formation in various epithelial cell lines. For example, Scheiffele et al., 1998 [13], who were using immunogold labeling, followed by electron microscopy, noticed that in the polarized epithelial cell line MDCK, Cav-1 and -2 are found together on basolateral caveolae, whereas the apical membrane, where only Cav-1 is present lacks caveolae. This observation suggests that Cav-1 and -2 heterooligomers, but not Cav-1 homooligomers, are essential for caveolar biogenesis in MDCK cells. A follow-up study revealed that the expression of Cav-1mutant, which prevented the formation of the large Cav-1/-2 hetero-oligomeric complexes, led to intracellular retention of Cav-2 and loss of caveolae, while overexpression of wild-type (WT) Cav-2 increased number of caveolae, thus supporting the role of Cav-2 in caveolar biogenesis in MDCK cells [14]. Independently, using freeze-fracture immunoelectron microscopy, Fujimoto and colleagues [15] have shown that coexpression of Cav-1 and Cav-2 resulted in a more efficient formation of deep caveolae than Cav-1 alone in hepatocellular carcinoma cell line (HepG2). Using electron microscopy, we have previously shown that in addition to Cav-1, Cav-2 is also essential for de novo assembly of caveolae in human prostate cancer cell line LNCaP [3]. Furthermore, the stimulatory effect of Cav-2 on caveolae assembly in LNCaP cells depended on the constitutive phosphorylation of Cav-2 at serine residues 23 and 36 by casein kinase 2 or a casein kinase 2-like kinase. Mutation of serine residues 23 and 36 to alanine, reduced the number of plasmalemma-attached caveolae and increased the accumulation of noncoated vesicles but did not affect trafficking of Cav-2 to caveolae and interaction with Cav-1 resulting in a formation of high molecular weight heterooligomeric complexes [3]. Although serine 23 phosphorylation did not affect caveolae assembly per se, it cooperated with serine 36 during caveolae biogenesis. Remarkably, S23A-Cav-2 increased the number of noncoated vesicles in proximity to plasma membrane, suggesting that serine 23 phosphorylation could be important for plasma membrane caveolae turnover and trafficking.

## 3. Serine 23 Phosphorylation as a Specific Marker of Cav-2 Targeting to Plasma Membrane Caveolae and Lipid Rafts in Epithelial and Endothelial Cells

Because in addition to plasma membrane caveolae and lipid rafts, Cav-2 can exist in other subcellular compartments such as ER, Golgi, cytosolic vesicles, lipid droplets, or nucleus, we have decided to examine if serine phosphorylation of Cav-2 depends on subcellular location. Using a combination of reconstituted epithelial cell-based systems such as LNCaP and FRT cells as well as endogenously expressing caveolins primary human endothelial cells, we have examined if phosphorylation of Cav-2 at serines 23 and 36 can be regulated by Cav-1 and subcellular targeting to noncaveolar intracellular compartments versus plasma membrane lipid raft/caveolae in the aforementioned epithelial and endothelial cells. Specifically, detergent insolubility and sucrose flotation gradients experiments revealed that Cav-2 was phosphorylated at serine 23 primarily in detergent resistant microdomains (DRMs). In contrast, serine 36 phosphorylation occurred in non-DRMs. Furthermore, immunofluorescence microscopy studies determined that in the presence of Cav-1 serine 23-phosphorylated Cav-2 localized mostly to plasma membrane, while serine 36-phosphorylated Cav-2 primarily resided in intracellular perinuclear regions. To directly address the role of Cav-1 in regulating phosphorylation of endogenous Cav-2, we have used siRNA approach. The specific knockdown of Cav-1 in human endothelial cells decreased Cav-2 phosphorylation at serine 23 but not serine 36. Thus, upregulation of serine 23 phosphorylation of Cav-2 depends on Cav-1-driven targeting to plasma membrane lipid rafts and caveolae [16].

## 4. Tyrosine 19 and 27 Phosphorylation of Cav-2: Regulation and Potential Role in Cell Signaling and Function in or out of Lipid Rafts/Caveolae?

Cav-2 has been shown to undergo Src-induced phosphorylation on tyrosine 19, using phosphotyrosine 19 (pY 19)-Cav-2-specific antibody in NIH-3T3 cells stably overexpressing c-Src. Stimulation of adipocytes with insulin resulted in increase in pY 19-Cav-2. Unlike total Cav-2, pY 19-Cav-2 primarily localized near focal adhesion sites. Using Gst-fused Cav-2, several Src homology 2 (SH2) domain containing proteins were identified, such as c-Src, Nck, and Ras-GAP to potentially interact with Cav-2 in a phosphorylation-dependent manner. These data suggest that tyrosine 19-phosphorylated Cav-2 could possibly be a docking site for SH2 domain containing proteins [17]. Interestingly, pY 19-Cav-2 remained associated with lipid rafts/caveolae, but no longer formed high molecular mass hetero-oligomeric complexes with Cav-1. Therefore, the authors concluded that phosphorylation of Cav-2 at tyrosine 19 may act as a signal dissociating Cav-2 from Cav-1 oligomers [17]. However, because Cav-2 requires Cav-1 for caveolar association, it is difficult to reconcile this concurrent lipid raft/caveolar association of pY 19-Cav-2 with a loss of interaction with Cav-1. Further studies may be required to thoroughly investigate this intriguing regulation. For example, coimmunoprecipitation experiments with Cav-1 and -2 antibodies could be performed to test if pY 19-Cav-2/Cav-1 interaction is indeed lost. 

 In addition to tyrosine 19, Cav-2 can also be phosphorylated at tyrosine 27. Remarkably, pY 27-Cav-2 appeared to be more critical than pY 19-Cav-2 for binding c-Src, Nck, and Ras-GAP. EGF stimulated Cav-2 phosphorylation at both tyrosines 19 and 27 in A431 cells; however, the temporal response to EGF was different; that is, Cav-2 was phosphorylated at tyrosine 19 in a rapid and transient fashion, whereas phosphorylation at tyrosine 27 was sustained. Interestingly, just as pY 19-, pY 27-Cav-2 also associated with lipid rafts/caveolae but did not form high molecular heterooligomeric complexes with Cav-1 [18].

## 5. The Negative Role of (Lipid Raft/Caveolar) Cav-2 in Regulating Lung Endothelial Cell Proliferation

The possibility for the involvement of Cav-2 in regulating endothelial cell proliferation and differentiation was suggested by the observation that Cav-2 KO mice develop a hyperproliferative phenotype in the lungs involving vascular endothelial growth factor receptor 2 (VEGFR-2 = Flk-1 in mouse) positive cells [12]. Because Flk-1 is widely believed to be predominantly expressed in endothelial cells, this observation suggests that Cav-2 may negatively regulate microvascular endothelial cell proliferation in the lung. However, due to the overall complexity of the in vivo system, it is impossible to unequivocally conclude if Cav-2 directly regulates lung microvascular endothelial cell proliferation. Therefore, we immunoisolated and characterized pure populations of lung endothelial cells from Cav-2 KO and wild-type (WT) mice and directly compared their proliferation potential and the expression or phosphorylation levels of cell cycle-associated signaling proteins. These studies determined that Cav-2 directly suppresses lung microvascular endothelial cell proliferation, possibly via inhibition of extracellular signal regulated kinase 1/2 (ERK1/2) phosphorylation, increased expression of cyclin-dependent kinase (cdk) inhibitors p16INK4 and p27Kip1, and activation (hypophosphorylation) of the retinoblastoma (Rb) protein, resulting in a decreased cell-cycle progression [19]. Recently, another group using a combination of miRNA, siRNA, and plasmid overexpression approaches has confirmed antiproliferative function of Cav-2 in rat prostate endothelial cells (YPEN-1) [20].

We are now investigating the mechanistic nature of this negative regulation by Cav-2. Since Cav-2 almost entirely targets to lipid rafts/caveolar microdomains in mouse lung endothelial cells used in our experiments [21], our data suggest that the inhibitory effect of Cav-2 on endothelial cell proliferation is most likely initiated in plasma membrane lipid rafts and caveolae. However, additional mechanistic studies will be required to unequivocally conclude if the latter is always true. 

How could the inhibitory signal be transduced from caveolar Cav-2 to downstream target proteins regulating endothelial cell proliferation? It may be either through the negative effect on some of many growth factor receptors that are present in caveolae or even via a direct effect on intracellular proteins involved in cell proliferation. For example, the N and to a lesser degree C-terminal ends of caveolae-localized Cav-2 face the cytosol and could potentially interact with signaling proteins located in cytosol, membrane vesicles, and other compartments which can come in a close proximity with caveolae.

## 6. (Lipid Raft/Caveolar) Cav-2 Suppresses the Transforming Growth Factor Beta-Induced Signaling and Inhibition of Mouse Lung Endothelial Cell Proliferation

Our most recent findings suggest that the role of Cav-2 in regulating lung microvascular EC proliferation is more complex and appears to be context specific [21]. Specifically, using a combination of WT and Cav-2 KO, along with retroviral re-expression approaches, we have determined that Cav-2 may be a physiological inhibitor of antiproliferative function and signaling of transforming growth factor beta (TGF-*β*) in mouse lung endothelial cells. Although treatment with TGF-*β* resulted only in a modest inhibitory effect on WT lung endothelial cells, it profoundly inhibited proliferation of Cav-2 KO lung endothelial cells. To confirm the specificity of the observed difference between WT and Cav-2 KO endothelial cells in response to TGF-*β*, we have stably re-expressed human Cav-2 in Cav-2 KO endothelial cells using a retroviral approach. Similar to WT endothelial cells, the antiproliferative effect of TGF-*β* was dramatically reduced in the Cav-2 re-expressing endothelial cells. This reduced antiproliferative effect of TGF- *β* in Cav-2 positive cells was demonstrated by three independent proliferation assays and correlated with a loss of TGF-*β*-mediated upregulation of p27 and subsequent reduction of the levels of hyperphosphorylated (inactive) form of the Rb protein in Cav-2 re-expressing endothelial cells. Mechanistically, Cav-2 inhibits antiproliferative action of TGF-*β* by suppressing Alk5/Smad2/3 pathway manifested by reduced magnitude and length of TGF-*β*-induced Smad2/3 phosphorylation as well as activation of Alk5/Smad2/3 target genes, plasminogen activator inhibitor-1 and collagen type I in Cav-2-positive endothelial cells. To examine possible changes in targeting of TGF-*β* receptors (T*β*Rs) or other components of TGF-*β* signaling pathway to lipid raft and caveolar domains, we performed detergent-free sucrose fractionation gradient. Our preliminary data suggest that expression of Cav-2 does not significantly change targeting of TGF-*β* receptors type I, Alk5 and Alk1 as well as Smad2/3 to caveolar and lipid raft microdomains. However, additional studies with control versus TGF-*β*-treated endothelial cells using various subcellular fractionation and immunofluorescence microscopy localization techniques will be needed for a more comprehensive analysis. One could possibly argue that perhaps the fact that Cav-1 expression levels are reduced at least by c.a. 50% in Cav-2 KO, relative to WT endothelial cells, could contribute to the enhanced antiproliferative effect of TGF-*β* in Cav-2 KO endothelial cells. This is, however, not the case, because the levels of re-expressed Cav-2 in Cav-2 KO endothelial cells were insufficient to significantly upregulate the expression levels of Cav-1 or change its targeting to lipid raft/caveolar microdomains. In addition, just as endogenous Cav-2 in WT endothelial cells, the re-expressed Cav-2 displayed normal targeting to plasma membrane and lipid raft/caveolae and did not affect subcellular targeting of endogenous Cav-1. Moreover, these low levels of re-expressed Cav-2 were adequate to reduce TGF-*β*-mediated responses in Cav-2 re-expressing cells to the levels that were comparable with WT endothelial cells [21]. Thus, the negative regulation of TGF-*β* signaling and function by Cav-2 is independent of both the expression levels and targeting of Cav-1 to lipid raft/caveolar microdomains. Further studies examining the detailed mechanisms responsible for inhibitory regulation of TGF-*β*-induced signaling and function in endothelial cells by Cav-2 will be necessary. For example, it will be important to examine for possible interactions of Cav-2 and Cav-1 with Alk5 or other components of TGF-*β* pathway in the absence and presence of TGF- *β*. In addition, it will be interesting to examine possible regulation of subcellular localization of Alk5, T*β*RII, or accessory receptors such as endoglin and *β*-glycan by Cav-2. Also, it will be important to examine the specific role of previously identified serine and tyrosine phosphorylation of Cav-2 [3, 16–18]. Finally, determining *in vivo* significance of our results, for example, how our findings may translate into a possible role of Cav-2 in regulating angiogenesis using various models of angiogenesis in which TGF-*β* pathway plays an important role, is clearly warranted.

## 7. Can (Lipid Raft/Caveolae-Localized) Cav-2 Be Considered as Molecular Switch in Controlling Mouse Lung Endothelial Cell Proliferation?

It is important to reconcile the role for Cav-2 in inhibiting TGF-*β*-mediated antiproliferative effect in endothelial cells [21] with previously described antiproliferative role of Cav-2 [19]. Although, in both cases, Cav-2 acts as an inhibitor, the final outcome depends on the specific context. Namely, in our previous studies, cell proliferation was evaluated only under optimal conditions, that is, in the absence of known growth inhibitors [19]. Under these conditions, Cav-2 dampens proproliferative effect of serum and growth factors, resulting in inhibition of endothelial cell proliferation. Conversely, in the presence of TGF-*β*, the role of Cav-2 could switch from anti- to proproliferative effect through the negative regulation of the growth inhibitory action of TGF-*β*/Alk5/Smad2/3 pathway [21]. Thus, it is plausible to suggest that Cav-2 protein present in caveolae could act as molecular switch counteracting excessive cell responses to both pro- and antiproliferative signals. Further studies will be necessary to solidify this newly proposed role for Cav-2 in endothelial and possibly other cell types.

## 8. Positive Role of (Noncaveolar) Cav-2 in Insulin-Stimulated Fibroblast Proliferation

Although in Cav-2 KO mice, adipose tissue remains normal and the protein levels of insulin receptor-*β* are not altered [22], the function of Cav-2 in cell-cycle regulation by insulin was examined in human insulin receptor-overexpressing rat 1 fibroblast (Hirc-B) cells, in which treatment with insulin induced Cav-2 gene expression in a time-dependent manner. Overexpression of Cav-1 in these cells resulted in inhibition of cell proliferation [23]. Furthermore, insulin-induced phosphorylation of Cav-2 on tyrosine 19, leading to increased interaction between Cav-2 and phospho-ERK. Interestingly, treatment with insulin resulted in translocation of phospho-ERK and pY19-Cav-2 to the nucleus. Downregulation of Cav-2 with siRNA suppressed the insulin-induced nuclear localization of ERK and attenuated the ERK-mediated c-Jun and cyclinD1 expression and DNA synthesis by insulin. Furthermore, insulin-induced interaction of Cav-2 with phospho-ERK was prevented when tyrosine 19 was mutated to alanine. In addition, actin cytoskeleton was shown to be involved in the nuclear translocation of Cav-2-ERK complex. Thus, the authors concluded that the tyrosine 19 phosphorylation of Cav-2 is required for actin cytoskeleton-dependent ERK nuclear import [24]. In subsequent study, the same group also reported that insulin-activated ERK is translocated to the nuclear envelope by Cav-2 and associates with lamin A/C in the inner nuclear membrane. Using site directed mutagenesis, they also determined that the Ser154-Val 155-Ser156 domain on the C-terminal of Cav-2 was essential for insulin—but not insulin growth factor-induced tyrosine 19 phosphorylation of Cav-2 and nuclear targeting of ERK and Cav-2 [25]. 

How could Cav-2 play an opposite role in regulating endothelial and fibroblast cell proliferation? The most likely reason is subcellular targeting. Although lipid raft/caveolar localization was not examined in HircB fibroblasts, unlike endothelial cells, HircB cells do not seem to express Cav-1 [23]. Thus, it is reasonable to suggest that caveolae are absent in these cells and due to lack of Cav-1, Cav-2 localization is limited to nonlipid raft intracellular compartments in the absence or even nucleus upon stimulation with insulin. It would be interesting to further elucidate these processes by comparing the effect of overexpressed Cav-2 in the absence versus the presence of Cav-1 and to examine if Cav-2 interacting with Cav-1 and thus localized to lipid raft/caveolar microdomains is able to possibly dissociate from Cav-1 and translocate to intracellular compartments, in particular nucleus in response to insulin.

## 9. Cav-2 and Human Cancer

The Cav-2 gene was colocalized with the Cav-1 gene to the locus D7S522 of human chromosome 7q31.1, a known fragile site (FRA7G) that is frequently deleted in human cancers, including squamous cell carcinomas of the head and neck, prostate cancers, renal cell carcinomas, ovarian adenocarcinomas, colon carcinomas, and breast cancers [26]. Although Cav-2 expression levels were unaltered by oncogenic transformation [27, 28], Cav-2 expression was upregulated in esophageal [29] and urothelial carcinoma [30]. Furthermore, the expression of Cav-2 was associated with shorter survival in stage I lung adenocarcinomas [31]. Cav-2 expression was also closely associated with basal-like immunophenotype and proved by univariate analysis to be a prognostic factor of breast cancer [32, 33]. Recently, the number of caveolae and the expression levels of Cav-2, Cav-1, and PTRF/cavin-1 were investigated in normal human prostate stromal, epithelial cells, androgen-dependent (LNCaP), and androgen-independent (PC3) cancer cell lines as well as in tissue obtained from patients with benign prostatic hyperplasia (BPH) and well-differentiated and poorly differentiated prostate cancer. These studies revealed that the number of caveolae was significantly reduced in LNCaP and PC3 cells. PTRF/cavin-1 expression levels were significantly reduced in both LNCaP and PC3 cells and in prostate cancer tissue. In contrast to PTRF/cavin-1 of which expression levels decreased, the expression levels of Cav-1 and -2 increased in prostate cancer tissue. Importantly, although both Cav-1 and -2 were nearly absent in androgen-dependent LNCaP cells, the expression levels of Cav-2 but not Cav-1 were markedly elevated in androgen-independent PC3 cells as compared to normal prostate epithelial cells. Overall, this data suggest that changes in the plasma membrane involving loss of caveolae and PTRF/cavin-1 expression occur during prostate cancer progression. Importantly, Cav-2 is upregulated during prostate cancer progression [34]. Since upregulation of Cav-2 in PC3 cells coincides with the absence of caveolae, this data suggests that noncaveolar Cav-2 could possibly play a positive role in prostate cancer progression. Since PC3 still express Cav-1, it is likely that Cav-2 still interacts with Cav-1 and targets to lipid rafts microdomains although direct studies examining the latter possibility will be required. Interestingly, a most recent study using siRNA and overexpression approaches demonstrated that Cav-2 could possibly differentially affect proliferation of various cell lines. For example, overexpression of Cav-2 in HepG2 hepatocellular carcinoma and siRNA knockdown in C6 glioma cell lines reduced cell proliferation. Furthermore, overexpression of Cav-2 in SH-SY5Y neuroblastoma and siRNA knockdown in HeLa epithelial cervical cancer and A549 lung adenocarcinoma cell lines promoted cell proliferation [35]. Little mechanistic insight is provided as to how Cav-2 could differentially regulate proliferation in the above mentioned cancer cell lines. However, studies focused on if and how the above reported differences depend on lipid raft/caveolar versus nonlipid raft/caveolar targeting of Cav-2 could shed more light.

## 10. Lipid Raft- versus Nonlipid Raft-Localized Cav-2 and Bacterial Invasion

Cav-2 has been shown to facilitate infection of murine lung epithelial cell line MLE-12 with *Pseudomonas aeruginosa *and that lipid raft targeting and tyrosine phosphorylation of Cav-2 as well as interaction with Csk and c-Src was important [36, 37]. Specifically, siRNA-mediated knockdown of Cav-2 decreased [36], while overexpression of WT but not Y19/27F-Cav-2 increased the ability of *P. aeruginosa *to invade MLE-12 cells. In addition, the siRNA knockdown of Cav-1 also resulted in reduction of *P. aeruginosa *invasion. However, unlike Cav-2 siRNA which did not change Cav-1 expression level, Cav-1 siRNA reduced Cav-2 expression levels, suggesting that Cav-2 rather than Cav-1 could be directly responsible [36]. Lipid raft dependence of *P. aeruginosa* invasion and Cav-2 phosphorylation was determined on the basis of various pharmacological inhibitors, including cholesterol affecting agents such as methyl-*β*-cyclodextrin or filipin [36, 37]. However, due to nonspecific nature of pharmacological inhibitors used in this study, it would be important to provide additional evidence for the specific involvement of lipid raft-localized Cav-2 in the process. For example, it would be vital to examine targeting of endogenous and overexpressed WT- and Y19/27F-Cav-2 to plasma membrane lipid rafts/caveolae before and at different stages of MLE-12 cell invasion with *P. aeruginosa* using sucrose floatation gradients and immunofluorescence microscopy. 

In another study, using siRNA approach, it was demonstrated that Cav-2, actin, E3 ubiquitin ligase, c-Cbl, and clathrin but not Cav-1 is involved in invasion of *Rickettsia conorii *in HeLa cells [38]. Considering that Cav-1 is required for lipid raft/caveolar targeting of Cav-2, the negative effect of Cav-1 siRNA could suggest that nonlipid raft/caveolar Cav-2 is responsible for facilitating invasion with this pathogen. 

 The role of noncaveolar Cav-2 was also suggested in chlamydial infection of various epithelial cell lines including HeLa and FRT cells, where Cav-2 associated with the chlamydial inclusion independently of Cav-1 [39]. However, the functional significance of Cav-2 either in the uninfected cell or in the chlamydial developmental cycle were not addressed in this study.

## 11. Cav-2, Lipid Droplets, and Metabolism

Lipid droplets are organelles that play important role in lipid metabolism and beyond [40]. It was shown that under certain conditions, Cav-2 can also be found in lipid droplets [41, 42]. Specifically, overexpressed Cav-2 accumulated not only in the Golgi apparatus but also in lipid droplets of the transiently transfected FRT cells [42]. In another study, it was also shown that Cav-2, especially its beta isoform, is targeted to the surface of lipid droplets by immunofluorescence and immunoelectron microscopy and by subcellular fractionation. Brefeldin A treatment induced further accumulation of Cav-2 along with Cav-1 in lipid droplets. Analysis of mouse Cav-2 deletion mutants revealed that the central hydrophobic domain (residues 87–119) and the NH_2_-terminal (residues 70–86) and COOH-terminal (residues 120–150) hydrophilic domains are all necessary for the targeting to lipid droplets. The NH_2_- and COOH-terminal domains appeared to be important to membrane binding and exit from endoplasmic reticulum, respectively, indicating that Cav-2 is synthesized and transported to lipid droplets as a membrane protein. In conjunction with findings that lipid droplets contain unesterified cholesterol and raft proteins, the result suggests that the lipid droplet surface may function as a membrane domain. It also suggests that lipid droplets could be related to trafficking of lipid molecules mediated by caveolins [41]. Although lipid metabolism is not the only function assigned to lipid droplets [Fujimoto and Parton], it is plausible to hypothesize that Cav2 present in lipid droplets could play some role in lipid storage or even lipoid metabolism. The question still remains as to what functional role could be associated with targeting of Cav-2 to lipid droplets besides potential involvement in lipid transport or metabolism? Which factor(s) is/are responsible for this preferred beta over alpha isoform of Cav-2 targeting to lipid droplets? Another question is if targeting of beta isoform of Cav-2 to lipid droplets plays a unique, possibly different role from that of alpha isoform. 

 As expected on the basis of lipid droplet targeting of Cav-2, later studies also implicated Cav-2 in regulating lipid metabolism due to changes in its expression levels associated with obesity and type 2 diabetes mellitus or adenoviral overexpression studies [43, 44]; however, the direct role of Cav-2 and its alpha versus beta isoforms in these processes remains still unclear.

## 12. Selected Examples of Other Cell Type-Specific Functions of Cav-2

Although originally only Cav-1 but not Cav-3 have been shown to interact and form high molecular weight heterooligomeric complexes with Cav-2 [45], later studies have shown that Cav-2 is coexpressed and may interact with Cav-3 in neonatal cardiac myocytes [46]. Interestingly, other findings show that although in fibroblasts Cav-2 interacts with Cav-1, but not -3, in myoblasts, all three caveolin isoforms could be coimmunoprecipitated [47]. This lack or presence of interaction for Cav-2 with Cav-3 in the above-mentioned cell types is interesting and may suggest that additional interacting partner(s) could be absent in fibroblasts but present in cardiac myocytes and myoblasts that could be responsible for such cell-specific differences. 

Cav-2 has also been shown to regulate endocytosis and trafficking of the M1 muscarinic acetylcholine receptor (mAChR) in MDCK cells [48]. The mechanism of this regulation appears somewhat complicated. Specifically, association of the plasma membrane M1 mAChR with Cav-2 inhibits receptor endocytosis through the clathrin-mediated pathway or retains the receptor in an intracellular compartment, resulting in attenuated receptor trafficking [48].

Cav-2 has also been shown to be required for apical lipid trafficking in the intestine of *Caenorhabditis elegans* [49].

Coimmunoprecipitation and fluorescence microscopic colocalization studies have revealed that Cav-2 interacts and colocalizes with Connexin 43 in rat epidermal keratinocytes, suggesting that both caveolins could regulate gap junctional intercellular communication [50].

## 13. What Can We Learn about Specific Contribution of Cav-2 to Lipid Raft/Caveolar-Mediated Cell/Tissue-Specific Signaling and Function from Studies with Cav-2 KO Mice?

Generation of and studies involving Cav-2 KO mice started revealing more information as to the pathophysiological significance of Cav-2. Initially, it was shown that Cav-2 KO mice develop hyperplasia in the lung associated with an increased number of Flk-1-positive, most likely endothelial cells [12], suggesting a role for Cav-2 in regulating lung endothelial cell proliferation and/or differentiation. As a consequence, Cav-2 KO mice are exercise intolerant, presumably due to an impaired gas exchange through the thickened alveolar septa. Interestingly, similar hyperproliferative phenotype was previously observed in Cav-1 KO mice [7]. However, the expression levels of Cav-2 in the lung of Cav-1 KO mice diminish to ca. 5% of the respective expression levels in WT mice. In contrast, the expression level of Cav-1 in the lung of Cav-2 KO mice is only reduced to 50%, and Cav-1 heterozygotes expressing comparable level of Cav-1 to that observed in Cav-2 KO mice do not develop hyperproliferative phenotype [12]. In addition, studies by Jasmin et al. 2004 have determined that similar to Cav-1 KO, STAT3 is hyperphosphorylated, and cyclin D1 and D3 levels were dramatically upregulated in the lungs of Cav-2 KO mice [51]. Overall, these findings implicate the lack of Cav-2 as the direct cause of the hyperproliferative phenotype observed in the lungs of both Cav-1 and Cav-2 KO mice. Moreover, since Cav-2 targeting to plasma membrane caveolae and lipid rafts depends on Cav-1, it is conceivable to suggest that proper targeting of Cav-2 to lipid raft/caveolar microdomains is necessary for Cav-2 to maintain physiological homeostasis in the lung by preventing the hyperproliferative phenotype and associated remodeling which can be found in the lungs of Cav-2 KO mice. 

Studies of Woodman et al. [52], revealed increased number of hematoxylin/eosin positive vessels in basic fibroblast growth factor-loaded matrigel plugs implanted to Cav-2 KO relative to WT mice. These data suggest that loss of Cav-2 could enhance basic fibroblast growth factor-induced angiogenesis in vivo. Additional studies examining the role of Cav-2 in postnatal angiogenesis will be required. 

Skeletal muscle abnormalities including tubular aggregate formation, mitochondrial aggregation, and increased numbers of M-Cadherin positive satellite cells that are skeletal muscle-specific stem/precursor cells were also reported in Cav-2 KO mice [53]. Because a similar phenotype was observed in Cav-1 KO mice and due to previously discussed dependence of Cav-2 on Cav-1, one could suggest that like in the lung, Cav-2, not Cav-1, is also directly responsible for maintaining normal phenotype in skeletal muscle. Again, targeting to lipid raft/caveolar microdomains should be essential for Cav-2 to perform this important physiological function. 

In another study, an increased number of Ki67-positive nuclei in the subventricular zone (SVZ) of Cav-2 KO brains could be observed, suggesting that Cav-2 may play a negative role in regulating proliferation of adult neural stem cells [54]. Similar data were obtained with Cav-1 KO mice, and since Cav-2 trafficking and localization to lipid rafts and caveolae depends on Cav-1 but not vice versa, as it is the case with pulmonary and muscle phenotypes, Cav-2 localizing to lipid rafts or caveolae could be directly involved in regulating adult neural stem cell proliferation. 

The most recent studies have reported increased sensitivity of Cav-2 KO but not Cav-1 KO mice to endotoxemia upon intraperitoneal injection with lipopolysaccharide (LPS) [55]. This augmented sensitivity of Cav-2 KO mice to LPS was associated with increased intestinal injury and intestinal permeability and correlated with enhanced expression of iNOS, production of nitric oxide (NO), and tyrosine 701 phosphorylation of STAT-1. In contrast to Cav-2, Cav-1 KO mice did not display an altered intestinal permeability and had decreased iNOS expression, NO production, and STAT-1 phosphorylation at tyrosine 701 compared to WT mice. Since Cav-2 is almost completely absent in Cav-1 KO mice, the authors concluded that not just the absence of Cav-2, but also the balance between Cav-1 and -2 is important for iNOS expression and ultimately for sepsis outcome [55]. Thus, unlike in previously reported role for Cav-2 in the lung, skeletal muscle, and adult neural stem cell proliferation, where Cav-2 played direct role, this new data also suggest that the ratio between the expression levels of Cav-1 and -2 could be the predominant factor responsible for the final outcome of the LPS challenge. 

Further studies will be required to precisely determine pathophysiological and mechanistic aspects of the reported phenotypes involving Cav-2 KO mice.

## 14. Conclusion and Future Directions

Multiple new findings from functional and mechanistic studies involving both in vivo and cell-based models have been recently reported, suggesting that one of the major protein components of plasma membrane caveolae, Cav-2, is not just an accessory protein, but it is also an important contributor to lipid raft- and caveolae-mediated signaling and function. The evidence continues to accumulate suggesting that in most cases in which loss or reduction of Cav-2 or Cav-1 results in a similar phenotype, Cav-2 plays a more direct role. The simple reason is that unlike Cav-1 which targets lipid rafts and caveolae on its own, interaction with Cav-1 is required for Cav-2 targeting to caveolae. Thus, whenever Cav-1 is lost or significantly reduced, Cav-2 loses its lipid raft/caveolar location and function, for which proper targeting of Cav-2 to lipid raft/caveolae is essential. In contrast, when Cav-2 is lost or reduced, Cav-1 still targets to lipid rafts/caveolae and retains all functions which do not depend on Cav-2. Despite considerable progress in the field, there are many questions to be answered. For example, what is the specific contribution of Cav-2 to lipid raft- and caveolae-mediated regulation of cell signaling and function? Exactly, how Cav-2 performs its functions in caveolae? Which specific domains of Cav-2 are mostly involved and what is the contribution of each phosphorylation site identified on Cav-2? Are there any signaling proteins of which targeting to caveolae depends more directly on Cav-2 than Cav-1 that could explain previously reported functions of Cav-2? What is the functional significance of serine 23 phosphorylation, which strictly depends on targeting of Cav-2 to caveolae in epithelial and endothelial cells? Are there additional posttranslational modifications of Cav-2 in addition to serine 23 which occur when Cav-2 reaches plasma membrane lipid rafts and caveolae? How important is tyrosine 19 and 27 phosphorylation or perhaps dephosphorylation of Cav-2 for the cell-specific role of lipid rafts/caveolae? How is it possible for tyrosine phosphorylated-Cav-2 to associate with caveolae/lipid rafts without being able to form high molecular heterooligomeric complexes with Cav-1? What are the interacting partners for Cav-2 necessary for inhibition of lung endothelial cell proliferation and TGF-*β*-induced signaling and function? What new phenotypes are waiting to be discovered in properly challenged Cav-2 KO mice and how they may help to better understand the contribution of Cav-2 to cell- and tissue-specific role of caveolae and lipid rafts?
